# Neuroleptic Malignant Syndrome: An Intensive Care Unit Case of Exceptionally High Creatinine Kinase and Myoglobin Levels

**DOI:** 10.7759/cureus.56306

**Published:** 2024-03-17

**Authors:** Eduardo Macedo, Filipa Rodrigues, Ana Rita Marques, Luís Ribeiro, Pedro Silveira

**Affiliations:** 1 Internal Medicine, Hospital de Braga, Braga, PRT; 2 Critical Care Medicine, Hospital de Braga, Braga, PRT

**Keywords:** acute renal failure, rhabdomyolysis, dopamine, neurologic emergency, antipsychotic medications, neuroleptic malignant syndrome

## Abstract

Neuroleptic Malignant Syndrome (NMS) is a rare, life-threatening neurologic emergency known to be related to the administration or sudden withdrawal of dopaminergic medications. The clinical course, symptoms, and bloodwork are very heterogeneous, making this syndrome difficult to identify. Thus, NMS is a diagnosis of exclusion. We present a case of severe NMS with exceptionally high creatinine kinase (CK) and myoglobin levels with unclear etiology and a challenging differential diagnosis. Also, our case stands out because it was serious, unique, and had a favorable outcome, which could contribute to the management of future similar cases.

## Introduction

Neuroleptic Malignant Syndrome (NMS) is a rare, life-threatening neurologic emergency caused by marked dysregulation of dopaminergic neurotransmission. It is known to be related to the administration or sudden withdrawal of dopaminergic medications. Incidence rates of this condition range from 0.02-3% among patients taking antipsychotic agents [1]. Although it most commonly affects young men, age is not a risk factor, as the syndrome described in most age groups [1,2]. The clinical course, symptoms, and bloodwork are very heterogeneous, making this syndrome difficult to identify and a diagnosis of exclusion [3].

We present a case of a 65-year-old man with unusually severe NMS with exceptionally high creatinine kinase (CK) and myoglobin levels who needed continuous invasive ventilation in an Intensive Care Unit (ICU) and who recovered after treatment with dantrolene, bromocriptine, and supportive care.

## Case presentation

An institutionalized 65-year-old man with a prior diagnosis of schizophrenia and hypertension presented in the emergency department (ED) with altered mental status. According to his caregivers, he had been agitating, clouded, and with hyperthermia two days before his admission. In that period, due to his agitation, haloperidol and levomepromazine were administrated more often than usual and his daily medications which included lorazepam, zotepine, carbamazepine, quetiapine and tapentadol were still administrated. His symptoms had progressed to mutism, muscular rigidity and, eventually, the patient became unresponsive, leading to his visit to the ED.

**Figure 1:**
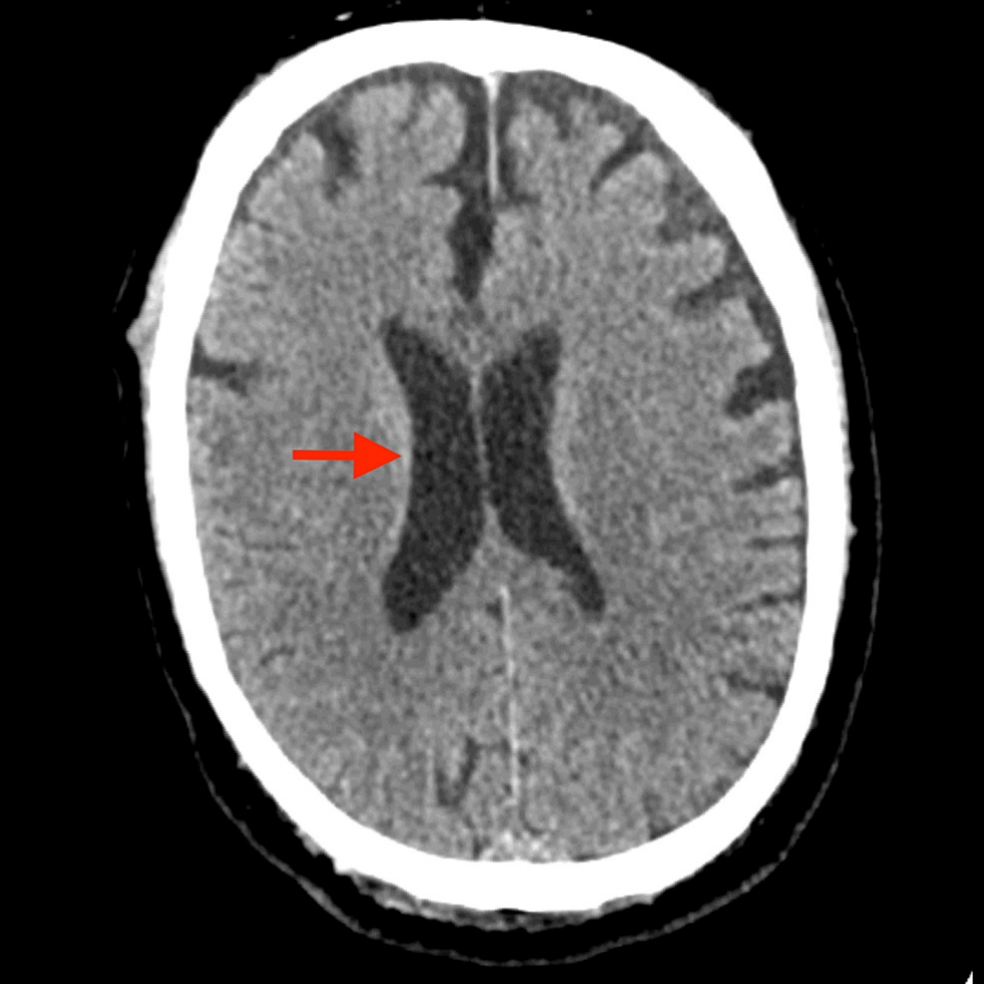
Axial section of cranial computed tomography scan Ventricular enlargement is characteristic of patients with Schizophrenia. It should be noted that there is no evidence of infection.

In the ED, the physical examination showed a patient with a depressed level of consciousness, conjugate gaze deviation to the right, patent airways, sustained tachycardia (140 bpm), severe hyperthermia (41ºC), and muscular rigidity. This raised suspicion of a convulsive crisis, and 5mg of diazepam was administered, with clinical improvement. Acute trauma workup was negative. Infections were excluded by performing a lumbar puncture and cranial-thoracic-abdominal-pelvic computed tomography scans (Figure 1). The bloodwork showed an acute renal failure with rhabdomyolysis. Due to clinical deterioration, sedation and orotracheal intubation were required and performed. NMS, malignant catatonia, and malignant hyperthermia were suspected, and the patient was admitted to the ICU for further workup.

**Figure 2:**
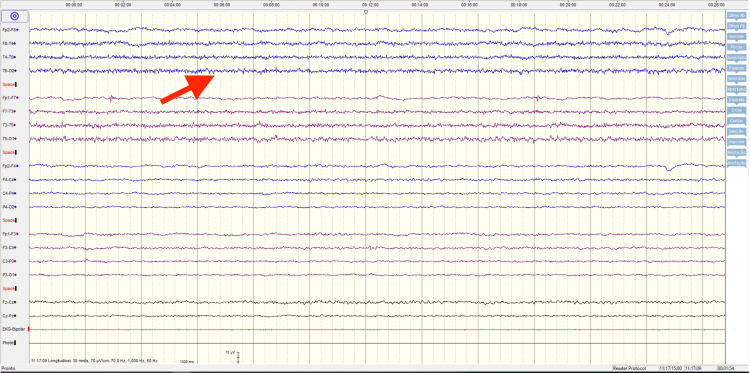
Electroencephalogram Encephalopathy likely related to sedative therapy. Exam without registration epileptiform activity.

In the ICU, the clinical and neurological evolution was slow but favorable. Initially, the rigidity and marked dyskinesias improved after 15 days of bromocriptine at a maximum dose (15mg every 8 hours) and dantrolene 90mg every 6 hours for 7 days. The improvement of rigidity was noted the day after the initiation of dantrolene therapy. Hyperthermia was very difficult to control, with the patient initially requiring paracetamol 1g every 8 hours, metamizole 2g every 8 hours, propranolol 20mg every 8 hours, and diclofenac in infusion. As there were concerns of epileptic activity, three electroencephalograms were performed. Even though no seizure activity was observed, it was empirically decided to medicate with levetiracetam 1g every 8 hours (Figure 2). Due to a prolonged orotracheal intubation (18 days), tracheostomy was necessary and performed percutaneously without complications. Severe rhabdomyolysis with extremely high creatinine kinase and myoglobin levels (967910 U/L and 843012 U/L respectively) led to acute kidney injury requiring continuous dialysis for 5 days.

By the time of discharge to an infirmary, the patient was tracheostomized, near his baseline level of activity, normothermic, with normal renal function, and without any signs of infection.

## Discussion

NMS is one of a group of acute dysautonomias that share common features with other entities such as serotonin syndrome, malignant hyperthermia, or malignant catatonia [1,4]. 

The pathophysiology of NMS is complex and poorly understood. There are some theories involving the central dopamine receptor blockade in the hypothalamus that may cause hyperthermia and dysautonomia, and interferences with nigrostriatal dopamine pathways that may cause rigidity and tremor [3,5-9]. Other neurotransmitter systems like epinephrine, serotonin, and acetylcholine also appear to be involved [10]. Alternative theories about disrupted modulations of the sympathetic nervous system and direct changes in muscle mitochondrial function as well as genetic predisposition to the disorder were proposed [11,12]. However, those theories are unable to explain all clinical manifestations, making NMS a disease of unknown cause.

NMS is defined by the association with drugs that block dopamine transmission and a tetrad of clinical features that include fever, rigidity, mental status changes, and autonomic instability [1]. The onset and duration of NMS is very variable. The mental status change is the initial symptom in 82% of patients, often taking the form of agitation [6]. Muscular rigidity is generalized and often extreme. Hyperthermia is a defining symptom and temperatures of more than 38ºC are typical. Autonomic instability is characterized by tachycardia in 88% of cases, followed by labile blood pressure, tachypnea, and diaphoresis [1]. Bloodwork findings often show profound CK elevation, which is typically more than 1000 international units/L [13]. Myoglobinuric acute renal lesions can result from rhabdomyolysis. Treatment always includes stopping the suspected causative agent and supportive care. Medical therapy includes the use of bromocriptine, dantrolene, and benzodiazepines. Electroconvulsive therapy can be an option, but it is generally reserved for patients not responding to other treatments [1]. Most episodes of NMS resolve in two weeks, but some cases persist for six months with residual symptoms reported, namely motor signs [14]. Mortality rates vary between 5-20% [15].

The differential diagnosis is a true challenge since several conditions may present with similar features. Serotonin syndrome has a similar presentation that is difficult to distinguish from NMS, but symptoms like rigidity and hyperthermia, when present, are less severe than in patients with NMS. Our patient had extreme rigidity and severe hyperthermia [1,16]. In malignant hyperthermia, the clinical appearance is quite similar to NMS, although symptoms are more fulminant and more common in cases of intense physical exercise [1]. Clinically, malignant catatonia is very similar to NMS and, therefore, difficult to distinguish. However, in this disease, the behavioral prodrome of some weeks is usually described. Also, laboratory values are more typically normal contrary to our patient, who had exceptionally high creatinine kinase and myoglobin levels [1].

Stopping causative agents, supportive care, and medical treatment are the three pillars of the management of patients with NMS. Removal of the causative agent is the single most important treatment in NMS. As our patient had lots of medication that was capable of causing the syndrome, these medications were immediately stopped. Mechanical ventilation, maintaining a euvolemic state through the help of dialysis, and controlling the fever and tremors was part of the supportive care. Dantrolene and bromocriptine were fundamental to improving his condition.

## Conclusions

The case that we present stands out for being difficult to diagnose, due to the extremely high muscle enzyme levels and for having a favorable outcome despite the odds. NMS can present with severe and varied symptoms that are crucial to detect. It is important to treat appropriately and early after keeping in mind the differential diagnoses. It is also worth mentioning the unknown cause of the case which can raise questions about which drugs cause the syndrome and whether doses and frequency of administration are important in its etiology. Understanding these questions will promote investigation to improve outcomes and mortality rates in patients diagnosed with this life-threatening syndrome.
